# Supplementary material for: Acceptance and compliance with micronutrient powder (MNP) among children aged 6–23 months in northern Nigeria
Source: PLOS Glob Public Health. 2022 Oct 17;2(10):e0000961. doi: 10.1371/journal.pgph.0000961 (PMC10022258; doi:10.1371/journal.pgph.0000961)
Supplement: S1 Dataset — (ZIP) [file pgph.0000961.s008.zip › S8_Dataset/Spot Checks.zip!/Spot Checks/110715_K_DW_AB_2_SC.b.pdf]

SPOT CHECK RECORDING FORM

State: Kebbi LGA: Danko Wasagu Community: Kangon Wasagu

Sex of index child: F   Age of index child (in months): 15months

Spot Check Information

1. **Date of MNP distribution to household** (circle option below based on LGA name above)**:**
   - **Kebbi:** BirninKebbi&Jega LGA: **June 16**
   - **Kebbi:** Danko-wasagu: **June 17**
   - **Adamawa:**Song, Ganye: **June 18**
   - **Adamawa:**Guyuk **June 19**
2. **Date of Spot Check Observation: 12/07/15**
3. **Number of sachets remaining**(Ask the caregiver to show you and count the specific number of sachets remaining)**:**

   39

4. **Number of sachets that should remainusing one MNP sachet per day** (Analyst only)**:**

   36

5. **Number of sachets remaining / number of sachets should be remaining**(Analyst only)**:**

   ____39_____ / ____36_____ = ____108.3_____ %

Probes

Question 1. You have **XX** MNP sachets remaining. Explain how easy or difficult it was to use the MNP product on a daily basis for your child 6 – 23 months? (Circle one answer)

Very Difficult **Difficult** **Easy** **Very Easy**

Question 2. Describe any specific challenges that you faced that made the MNP difficult to use on a daily basis?

Question 3. Describe specific aspects of the MNP product that you or your child enjoyed that made it easier to use on a daily basis?

It is very easy because once it is opened, it just to mix with the available food around such as fura, wake, faten wake e.t.c. and it is tasteless and colorless, so the child is not aware there is anything in the food, therefore the child is willing to take any food given her because she eats a lot now and she is very active and strong also

Question 4. What questions do you have about the MNP based on your experience using it?

No questions foe now in the usage of the product

Question 5. What specific recommendations do you have that we can do as a program to make the MNP easier to use on a daily basis?

You should give us something that can store the MNP to avoid losing the product also, it should be a continuous process as there are new born babies everyday
